# Seroprevalence of human coronaviruses among patients visiting hospital-based sentinel sites in Uganda

**DOI:** 10.1186/s12879-021-06258-6

**Published:** 2021-06-16

**Authors:** Elijah Nicholas Mulabbi, Robert Tweyongyere, Fred Wabwire-Mangen, Edison Mworozi, Jeff Koehlerb, Hannah Kibuuka, Monica Millard, Bernard Erima, Titus Tugume, Ukuli Qouilazoni Aquino, Denis K. Byarugaba

**Affiliations:** 1 College of Veterinary Medicine, Animal Resources and Biosecurity, Makerere University, Kampala, Uganda; 2 School of Public Health, Makerere University, Kampala, Uganda; 3 Mulago National Referral Hospital, Kampala, Uganda; 4 Makerere University Walter Reed Project, Kampala, Uganda

**Keywords:** Human coronaviruses, Febrile illnesses, Emerging viral infections, Seroprevalence

## Abstract

**Background:**

*Human coronaviruses are causative agents of respiratory infections with several* subtypes being prevalent worldwide. They cause respiratory illnesses of varying severity and have been described to be continuously emerging but their prevalence is not well documented in Uganda. This study assessed the seroprevalence of antibodies against the previously known human coronaviruses prior 2019 in Uganda.

**Methods:**

A total 377 serum samples collected from volunteers that showed influenza like illness in five hospital-based sentinel sites and archived were analyzed using a commercial Qualitative Human Coronavirus Antibody IgG ELISA kit. Although there is no single kit available that can detect the presence of all the circulating coronaviruses, this kit uses a nucleoprotein, aa 340–390 to coat the wells and since there is significant homology among the various human coronavirus strains with regards to the coded for proteins, there is significant cross reactivity beyond HCoV HKU-39849 2003. This gives the kit a qualitative ability to detect the presence of human coronavirus antibodies in a sample.

**Results:**

The overall seroprevalence for all the sites was 87.53% with no significant difference in the seroprevalence between the Hospital based sentinel sites (*p* = 0.8). Of the seropositive, the age group 1–5 years had the highest percentage (46.97), followed by 6–10 years (16.67) and then above 20 (16.36). An odds ratio of 1.6 (CI 0.863–2.97, *p* = 0.136) showed that those volunteers below 5 years of age were more likely to be seropositive compared to those above 5 years. The seropositivity was generally high throughout the year with highest being recorded in March and the lowest in February and December.

**Conclusions:**

The seroprevalence of Human coronaviruses is alarmingly high which calls for need to identify and characterize the circulating coronavirus strains so as to guide policy on the control strategies.

## Background

Human coronaviruses are common causative agents of respiratory infections with several subtypes being prevalent in many parts of the world. Coronaviruses are a complex group of viruses of the subfamily *Coronavirinae* in the family Coronaviridae of the order Nidovirales [1, 2]. This subfamily has four genera: Alphacoronaviruses, Betacoronaviruses, Gammacoronaviruses and Deltacoronaviruses, of which Alphacoronaviruses (HCoV-229E and HCoV-NL63) and Betacoronaviruses (HCoV-HKU1, SARS-CoV-1, SARS-CoV-2 HCoV-OC43 and MERS-CoV) infect humans [3, 4]. They are enveloped with a linear, non-segmented, positive sense, single-stranded RNA genome ranging between 27 kb to 32 kb which shows that they are the largest among the RNA viruses [5, 6]. Although they are phenotypically and genotypically diverse, they possess a common genomic organization with the replicase gene occupying two thirds of 5′ end of the genome in which two overlapping large open reading frames, ORF1a and ORF1b are found [5, 7].

Human coronaviruses were first discovered in the 1960s as causative agents of self-limited upper respiratory tract infections and until 2002, they were known to cause mild infections but this changed with the emergence of Severe Acute Respiratory Syndrome Coronavirus (SARS-CoV) [8, 9]. To date there are seven known Human coronaviruses (HCoVs) that have been identified, these are HCoV-229E, HCoV-NL63, HCoV-OC43, HCoV-HKU1, SARS-CoV, MERS-CoV and the newest discovered, SARS-CoV-2 [4, 10]. The first four HCoVs have been well known to be of worldwide distribution causing approximately 33.3% of human common cold infections [11]. However in some cases, these can cause severe illness in the elderly, children and all immunocompromised persons and patients especially those with underlying infections medical conditions like diabetes, hypertension, tuberculosis and AIDs [12, 13]. SARS-CoV suddenly emerged in Guangdong Province of China in 2002 with a fatality rate of 10–11%, causing severe pneumonia characterized by fever, headache and cough but later develops into life threatening respiratory failure and distress [14]. After 10 years, in 2012, MERS-CoV appeared in Saudi Arabia causing severe human respiratory disease with clinical presentation similar to SARS-CoV but with a higher fatality rate of 35% [15, 16]. Currently the world is facing a pandemic caused by a novel human coronavirus that started from the Hubei Province in China and now has spread the whole world, this novel coronavirus that had been previous named 2019-nCoV is now known as SARS-CoV-2 due to its similarity to the symptoms induced by SARS-CoV [17–19]. The transmission of SARS-CoV-2 to humans through an intermediate host has not been proven, although direct transmission from bats without the participation of an intermediate host may not be discarded. Nevertheless, studies have showed that human-to-human transmission through droplets and direct contact has been the most important mode of transmission to regions outside Hubei especially by asymptomatic carriers traveling from one area to another [20, 21].

Seroprevalence studies are important in understanding the prevalence of subclinical human coronavirus infections and the population’s herd immunity against these viruses. The Seroprevalence of human coronaviruses varies greatly among studies because of the different antigens, methodologies used, age and other demographic characteristics of the population studied [22]. The Seroprevalence estimates for Human coronaviruses range from 5 to 30% of all respiratory infections with up to 21.6% of the general population having serum antibodies [23, 24]. The Seroprevalences of the zoonotic human coronaviruses (SARS, MERS and SARS-2) have been reported to be below 6% in humans especially among those who do not come into contact with the intermediate hosts for these viruses [25–29]. Such results suggest that unknown asymptomatic and subclinical infections or unrecognized cases might exist in the general population that can underscore the role of human-to-human transmission [30–32].

Human coronavirus infections are commonly diagnosed by polymerase reaction using cDNA synthesized from RNA extracts from respiratory tract samples. However, for the establishment of exposure rates among the population, seroepidemiological studies offer an important avenue for painting a picture on HCoV infections. Coronaviruses, just like other respiratory infections show seasonal occurrence that correspond to the variation in climatic conditions of an area this is because climatic conditions affect the stability of the viruses which consequently affects their transmission and the degree of threat caused to humans [33]. Several studies have been done to establish the immune cross-reactivity among seasonal human coronaviruses (HKU1, OC43, 229E and NL63) and SARS-CoV-2 and these have revealed that this cross-reactivity is not protective against SARS-CoV-2 [34, 35]. Although an increasing threat of zoonosis and emerging pandemics caused by coronaviruses with the capacity to infect humans is now more obvious than ever, there is little known about their seroprevalence in most developing countries especially Uganda. Here we report the seroprevalence of human coronavirus antibodies in hospital-based surveillance sentinel sites in Uganda before the global pandemic of SARS-CoV-2.

## Methods

All methods used in the study were carried out in accordance with relevant guidelines and regulations.

### Study setting

**Fig. 1:**
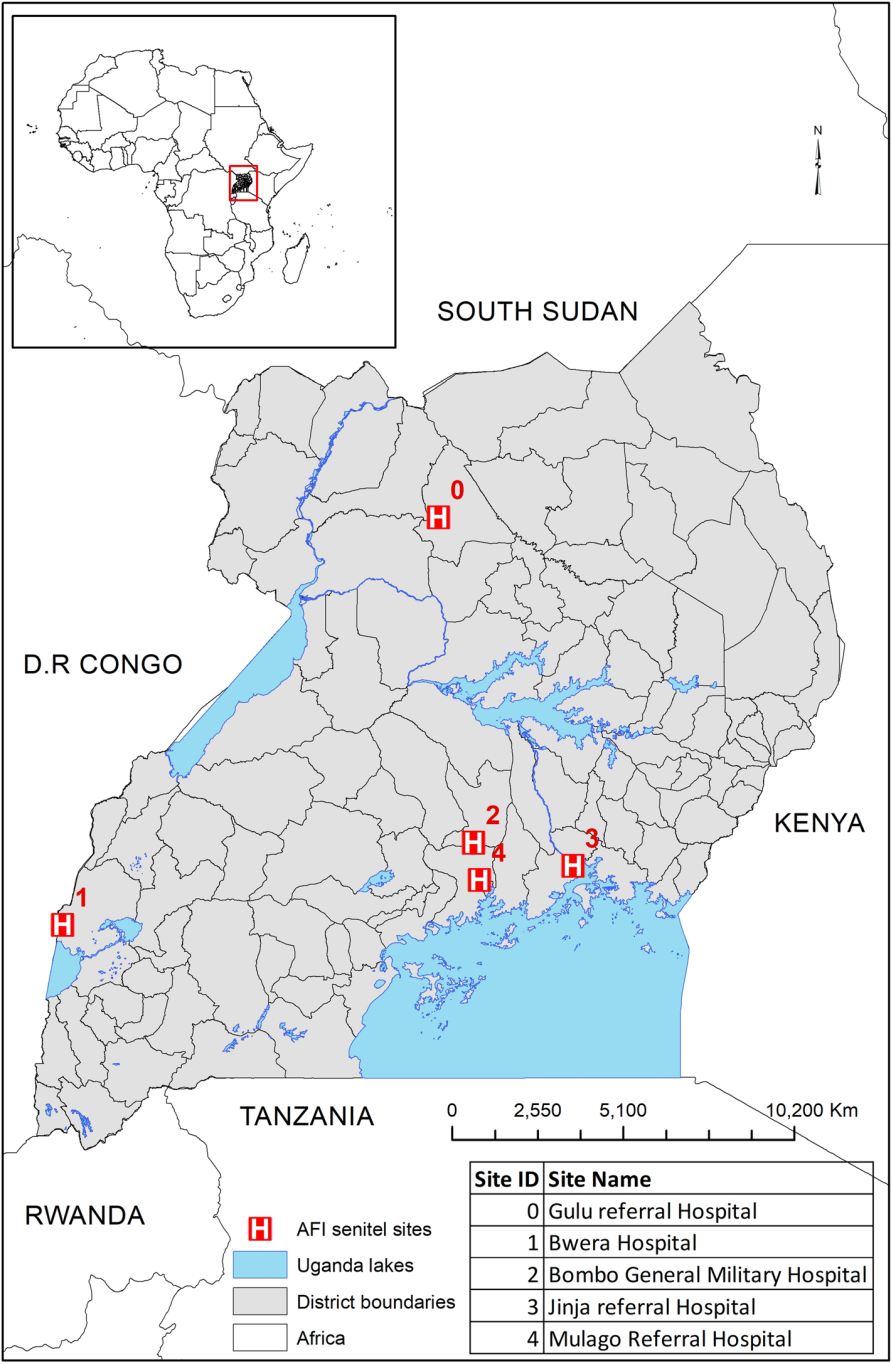
Map showing the Hospital based surveillance Sentinel sites in Uganda

The study was conducted among the Makerere University Walter Reed project established hospital-based sentinel sites for surveillance activities on acute febrile illnesses. Samples were obtained from the five hospital-based sites were established for surveillance which include Jinja (Eastern Uganda), Mulago (Central Uganda, Capital), Bwera (Western Uganda), Gulu (Northern Uganda) and Bombo Military hospital (Central Uganda) (Fig. 1).

### Sample collection

Patients reporting to these Sentinel sites between in 2018, aged 6 months or older presenting influenza like illness (ILI) were enrolled after written and signed consent for the adults while for minors, written and signed consent was obtained from their parents or legal guardians. Blood samples were drawn from the volunteers into anticoagulant free vacutainers then transported to the Emerging Infectious Diseases Laboratory, Makerere University. The blood was allowed to clot by leaving it standing at room temperature for 60 min, then centrifugation was done at 1500 x g for 10 min to the serum. The serum samples were kept at -80 °C and analyzed in 2019.

### Case definition

An ILI patient was defined as any individual having a fever of temperature 38 °C and above, plus any of the following clinical signs (cough, sore throat, myalgia and headache) for a period not exceeding 10 days according to the established guidelines by WHO with modifications in other studies [36]. Demographic characteristics of the patients were recorded on a standardized form.

### Sample size determination

The samples size was computed using the formula adapted from Veterinary Epidemiology by Michael Thrusfield at 95% confidence interval, prevalence of 50% (Xiuping et al., 2007) and precision of 5%.

$$ n=\frac{1.96^2x\ {P}_{exp}\left(1-{P}_{exp}\right)}{d^2} $$

n is the required sample size, P_exp_ is expected prevalence, d is desired absolute precision

$$ n=\frac{1.96^2x\ 0.5\left(1-0.5\right)}{0.05^2}=384 $$

Due to resource limitations, a total of 377 samples were randomly selected from 1485 samples that had been collected from the Sentinel sites between in 2018.

### Laboratory testing and analysis

The archived serum samples were retrieved, thawed to room temperature and tested using a commercial Qualitative Human Coronavirus IgG ELISA kit Cat. No. MBS9301037 (renamed HCoV-HKU-IgG ELISA kit) from MyBioSource. The kit uses a nucleoprotein, aa 340–390 to coat the wells and according to the manufacturer, since there is significant homology among the various human coronavirus strains with regards to the coded for proteins, there is significant cross reactivity beyond SARS-CoV HKU-39849 2003 [37]. This gives the kit a qualitative ability to detect the presence of human coronavirus antibodies in a sample. The assay was done according to manufacturer’s recommendation. Briefly, the reagents and samples were brought to room temperature, the positive and negative control wells as well as the sample wells were set. 50 μl each of the positive control, negative control and undiluted samples were added to respective wells. 100 μl of HRP- conjugate reagent was added to all the wells, covered with an adhesive strip and then incubated for 60 min at 37 °C. The plates were then washed four times after which 50 μl of Chromogen Solution A and 50 μl of Chromogen Solution B was added to each well successively, mixed gently and incubated for 15 min at 37 °C when protected from light. 50 μl of stop solution was added to each well and then Optical Density read at 450 nm using an ELISA reader at 5 min after the addition of the stop solution. The cut off for positivity were calculated per assay as the average OD value for the negative control wells + 0.15.

### Statistical analysis

We categorized volunteers in two broad groups (below 5 years and those above 5 years) to calculate the odds for seropositivity according to age group of volunteers. The computation was done manually using the formula adapted from Veterinary Epidemiology by Michael Thrusfield at 95% confidence interval.

## Results

### Demographic and clinical characteristics of enrolled volunteers

The demographic profile of the samples is summarized in Table 1. Majority of the samples (45%) were from volunteers of 1–5 years of age and were gender balanced (48.8% males, 51.2% females).

**Table 1:** Demographic characteristics of the samples

Demographic Characteristic	Numbers (%)
Gender	
Male	184 (48.8%)
Female	193 (51.2%)
Age in years	
< 1	28 (7.4%)
1–5	171 (45.4%)
6–10	68 (18.0%)
11–15	27 (7.2%)
16–20	22 (5.8%)
> 20	61 (16.2%)
occupation	
Pre-school Children	160 (42.4%)
Schooling /Students	139 (36.9%)
Employed	30 (8.0%)
Unemployed	48 (12.7%)

### Seropositivity among the samples

Of the 377 serum samples analyzed, 330 (87.53%) were seropositive while 47 (12.47%) were seronegative (Table 2). There was no significant difference in the seroprevalence of Human Coronavirus antibodies in the different sentinel sites (95% CI, *p*-value =0.8).

**Table 2:** Seroprevalence in different sites

Site	Seropositive Positive	Seronegative Negative	Seroprevalence (%)
Gulu (*n* = 76)	64	12	84.21
Mulago (*n* = 79)	64	15	81.01
Jinja (*n* = 68)	66	2	97.06
Bwera (*n* = 76)	70	6	92.16
Bombo (*n* = 78)	66	12	84.62
**Over all (*n*** **= 377)**	**330**	**47**	87.53

When we examined the seroprevalence according to age groups (Table 3), age group 1–5 years had the highest seroprevalence 90.6% with 47% of the seropositives falling in this age group. Further, 63% of all the seropositives were in the age bracket 1–10 years which also constituted 63% of all the samples. The percentage of the seropositive samples for each age was high with a mean of 86.63% and standard mean error 1.029.

**Table 3:** showing seroprevalence according to groups

Age group years (n)	Seropositive samples	% Seropositive within age group	% Seropositive in total samples
< 1(*n* = 28)	24	85.71	7.27
1–5 (*n* = 171)	155	90.64	46.97
6–10 (*n* = 68)	55	80.88	16.67
11–15 (*n* = 27)	23	85.19	6.97
16–20 (*n* = 22)	19	86.36	5.76
20 < (*n* = 61)	54	88.52	16.36
Total (377)	330	87.53	

The volunteers were divided into two broad categories, those below 5 years of age and those above 5 years to calculate the odds ratio for seropositivity (Table 4), an odds ratio of 1.6 was obtained (CI 0.863–2.97, *p* = 0.136). This shows that those below 5 years are more likely to be seropositive compared to those above 5 years.

**Table 4:** showing the broad age categories

Age category	Seropositive samples	Seronegative samples	Odds
Below 5 (199)	179 (89.9%)	20 (10.1%)	8.95
above 5 (178)	151 (84.8%)	27 (15.2%)	5.59

### Seasonality of the seropositivity among the volunteers

**Fig. 2:**
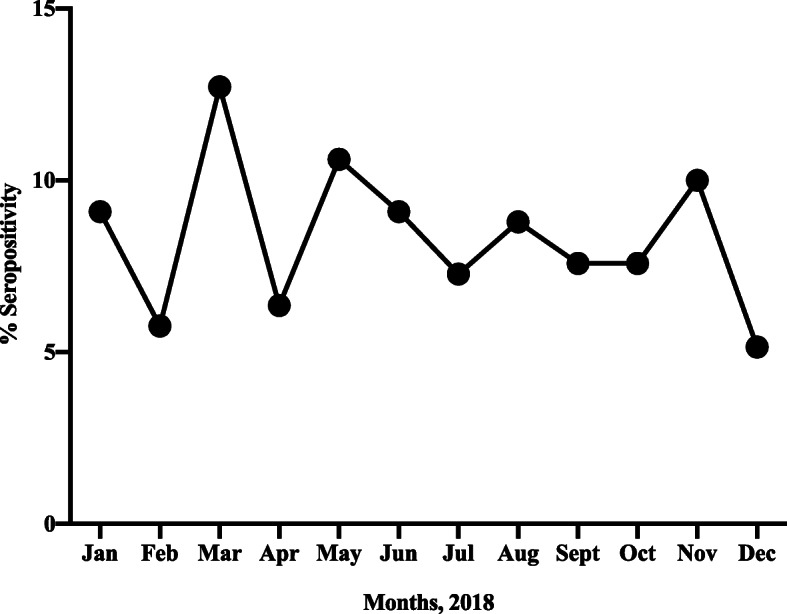
showing the variation of percentage seropositivity across the year (2018)

The seropositivity was generally high throughout the year with highest being recorded in March and the lowest in February and December as shown in Fig. 2.

## Discussions

Human coronaviruses are known to have a wide distribution and endemic to most countries in the world but usually limited information is available on their presence and circulation in Sub-Saharan countries like Uganda. Conducting serosurvey studies is important in establishing baseline knowledge of endemic viruses in a community especially examining the extent of exposure of the population to coronaviruses. We report on the level seroprevalence of human coronaviruses in hospital-based sentinel sites in Uganda at a time when country and the world at large is facing a pandemic caused by SARS-CoV2. This will provide a picture about the possible level of exposure and population-based immunity against human coronaviruses.

The study observed a high seroprevalence (87.5%) of human coronavirus antibodies in volunteers that visited the hospital-based sentinel sites. This high seroprevalence was consistent with other studies elsewhere that showed a prevalence of antibodies to four human coronaviruses (229E, OC43, NL63 and HKU 1) to be above 90% [38, 39]. This is still consistent with other studies that have shown that human coronaviruses have a global distribution [7, 40]. Although in our study we did not screen for specific strain of human coronavirus, the result shows a great exposure of the volunteers to human coronaviruses. The hospital-based sentinel sites from which the samples were collected are major referral health centers in the respective regions in the country, thus this result give an overall picture of the seroprevalence of human coronavirus antibodies in the country. A previous surveillance study in hospital-based sites in Uganda showed 1.5% PCR positivity for human coronaviruses which further proves that these viruses circulate within the population [41].

The qualitative Human coronaviruses antibody IgG ELISA kit that was used had its wells coated with a nucleoprotein, aa 340–390 (SARS corona HKU-39849) [37]. There is no commercially available kit which can universally detect antibodies against all circulating coronaviruses but given that there is significant homology among the various human coronavirus strains with regards to the coded for proteins, there is significant cross reactivity beyond SARS-CoV HKU-39849 2003 [23]. This gives the kit a qualitative ability to detect and give an overall picture of the seroprevalence of human corona antibodies in the volunteers. IgG antibodies are known to be detected 4 days after onset of disease and may persist in patients for a period of 36 months in individual after recovery [42]. Never the less, this suggests that this observed high seroprevalence among volunteers could be due to a recent or earlier exposure to the human coronaviruses. Without corresponding PCR or culture experiments for the tested samples, it was not possible to confirm the presence and the infectiveness of the virus in the volunteers.

In this study, seropositivity was highest among children between 0 and 5 years compared to other age group ranges. The samples were selected completely at random therefore the numbers per age group is not equal which limits our degree of comparison. However, it is true that the seroprevalence of human coronaviruses antibodies vary greatly depending on the age of the population [22, 43]. The computed odds ratios show that children under 5 years are more likely to be seropositive compared to individuals above 5 years of age. This agrees with the study which suggested that primary exposure to and infection by HCoVs takes place in childhood [7, 44–47]. The first years of an individual usually sees them transit from purely parent protection and isolation to open exposure in school settings. It is probable that this exposure makes this age group more susceptible to contracting human coronaviruses resulting in the high seropositivity.

In terms of seasonality, the seroprevalence was generally constant throughout the year with slight variations observed. The highest seroprevalence was observed in March and the lowest in February and December. Although no data about climate was analyzed during this study, Uganda experiences little annual variation in temperature with the average coolest months between June and September, while the rainy seasons occur between March and June, then between September and November, notwithstanding that the country experiences occasional light rains almost through the year especially for regions around Lake Victoria [48]. These weather conditions seem to favor the circulation of human coronavirus which elsewhere have been documented to circulate more frequently during winter season [11]. In addition, Studies in some countries in Africa (South Africa, Ghana and Kenya) have also shown that human coronaviruses circulate continuously throughout the year [49–51]. All these are in line with the argument that the activity of human coronaviruses is sporadic throughout the year with no clear seasonality [52] unlike influenza whose seasonality in Uganda has been established [53]. With the current spread of SARS-CoV-2 across the globe, seasonal differences have been preliminary suggested to might have contributed to the spread and severity of COVID-19, with countries in the Northern hemisphere being more affected than those in the Tropical regions and Southern hemispheres [54]. This therefore points to the need of continuously doing serosurveillance over a longer period of time to establish and describe the seasonality of Human coronaviruses in Uganda in order to minimize the disease burden caused.

Serological cross-reactivity between pre-COVID-19 human coronaviruses and SARS-CoV-2 antibodies has been shown with the nucleocapsid protein but this is not surprising because this protein is conserved and shows cross reactivity among human coronaviruses [34]. Cross reactivity between the spike protein of SARS-CoV-2 and that of other previously circulating HCoVs is limited because this protein is strain specific especially the S1 domain of the spike protein [34, 55]. The importance of these SARS-CoV-2 cross-reactive antibodies in the population towards protection against the infection is not yet fully studied and thus cannot be related to this observed high seroprevalence of HCoVs and the limited deaths in Uganda compared to other countries.

This study had limitations. The sentinel sites are not evenly distributed in the country, the lack of measurement of climatic factor to relate with the observed seroprevalence. Since the kit used was not quantitative in nature, we did not establish the antibody titer in the samples which could be correlated with the time of infection.

## Conclusion

This study reports the high seroprevalence of antibodies against Human coronaviruses. Human coronaviruses are important emerging pathogens and currently the world is facing a devastating pandemic caused by SARS-2, there is therefore need for continuous viral surveillance. There is need to determine whether these antibodies possessed due to previous exposure to coronaviruses can offer protection to these emerging viral strains.

## Data Availability

All data generated is available from the corresponding author on reasonable request.

## Abbreviations

HCoV: Human Coronavirus
SARS: Severe Acute Respiratory Syndrome
MERS: Middle East Respiratory Syndrome
cDNA: complementary DNA

## References

[1] Gorbalenya AE, Enjuanes L, Ziebuhr J, Snijder EJ (2006). Nidovirales: evolving the largest RNA virus genome. Virus Res.

[2] Masters PS (2006). The molecular biology of coronaviruses. Adv Virus Res.

[3] McBride R, Fielding BC (2012). The role of severe acute respiratory syndrome (SARS)-coronavirus accessory proteins in virus pathogenesis. Viruses..

[4] Lim YX, Ng YL, Tam JP, Liu DX (2016). Human coronaviruses: a review of virus–host interactions. Diseases..

[5] Lai MM, Cavanagh D (1997). The molecular biology of coronaviruses. Adv Virus Res.

[6] Brian D, Baric R (2005). Coronavirus genome structure and replication. Coronavirus replication and reverse genetics: Springer.

[7] Zhou W, Wang W, Wang H, Lu R, Tan W (2013). First infection by all four non-severe acute respiratory syndrome human coronaviruses takes place during childhood. BMC Infect Dis.

[8] Consortium CSME (2004). Molecular evolution of the SARS coronavirus during the course of the SARS epidemic in China. Science..

[9] Memish ZA, Cotten M, Meyer B, Watson SJ, Alsahafi AJ, Al Rabeeah AA (2014). Human infection with MERS coronavirus after exposure to infected camels, Saudi Arabia, 2013. Emerg Infect Dis.

[10] Lai C-C, Shih T-P, Ko W-C, Tang H-J, Hsueh P-R (2020). Severe acute respiratory syndrome coronavirus 2 (SARS-CoV-2) and corona virus disease-2019 (COVID-19): the epidemic and the challenges. Int J Antimicrob Agents.

[11] van der Hoek L (2007). Human coronaviruses: what do they cause?. Antivir Ther.

[12] Pene F, Merlat A, Vabret A, Rozenberg F, Buzyn A, Dreyfus F, Cariou A, Freymuth F, Lebon P (2003). Coronavirus 229E-related pneumonia in immunocompromised patients. Clin Infect Dis.

[13] Gorse GJ, O’Connor TZ, Hall SL, Vitale JN, Nichol KL (2009). Human coronavirus and acute respiratory illness in older adults with chronic obstructive pulmonary disease. J Infect Dis.

[14] Graham RL, Donaldson EF, Baric RS (2013). A decade after SARS: strategies for controlling emerging coronaviruses. Nat Rev Microbiol.

[15] Zaki AM, Van Boheemen S, Bestebroer TM, Osterhaus AD, Fouchier RA (2012). Isolation of a novel coronavirus from a man with pneumonia in Saudi Arabia. N Engl J Med.

[16] Kim Y, Cheon S, Min C-K, Sohn KM, Kang YJ, Cha Y-J (2016). Spread of mutant Middle East respiratory syndrome coronavirus with reduced affinity to human CD26 during the south Korean outbreak. MBio..

[17] Li Q, Guan X, Wu P, Wang X, Zhou L, Tong Y, Ren R, Leung KSM, Lau EHY, Wong JY, Xing X, Xiang N, Wu Y, Li C, Chen Q, Li D, Liu T, Zhao J, Liu M, Tu W, Chen C, Jin L, Yang R, Wang Q, Zhou S, Wang R, Liu H, Luo Y, Liu Y, Shao G, Li H, Tao Z, Yang Y, Deng Z, Liu B, Ma Z, Zhang Y, Shi G, Lam TTY, Wu JT, Gao GF, Cowling BJ, Yang B, Leung GM, Feng Z (2020). Early transmission dynamics in Wuhan, China, of novel coronavirus–infected pneumonia. N Engl J Med.

[18] Gorbalenya AE (2020). Severe acute respiratory syndrome-related coronavirus–the species and its viruses, a statement of the coronavirus study group. BioRxiv..

[19] Chen N, Zhou M, Dong X, Qu J, Gong F, Han Y, Qiu Y, Wang J, Liu Y, Wei Y, Xia J', Yu T, Zhang X, Zhang L (2020). Epidemiological and clinical characteristics of 99 cases of 2019 novel coronavirus pneumonia in Wuhan, China: a descriptive study. Lancet.

[20] Chang D, Lin M, Wei L, Xie L, Zhu G, Cruz CSD (2020). Epidemiologic and clinical characteristics of novel coronavirus infections involving 13 patients outside Wuhan, China. Jama.

[21] Carlos WG, Dela Cruz CS, Cao B, Pasnick S, Jamil S (2020). Novel Wuhan (2019-nCoV) coronavirus. Am J Respir Crit Care Med.

[22] Severance EG, Bossis I, Dickerson FB, Stallings CR, Origoni AE, Sullens A, Yolken RH, Viscidi RP (2008). Development of a nucleocapsid-based human coronavirus immunoassay and estimates of individuals exposed to coronavirus in a US metropolitan population. Clin Vaccine Immunol.

[23] Woo PC, Lau SK, Chu C-M, Chan K-H, Tsoi H-W, Huang Y (2005). Characterization and complete genome sequence of a novel coronavirus, coronavirus HKU1, from patients with pneumonia. J Virol.

[24] Chan C, Tse H, Wong S, Woo P, Lau S, Chen L (2009). Examination of seroprevalence of coronavirus HKU1 infection with S protein-based ELISA and neutralization assay against viral spike pseudotyped virus. J Clin Virol.

[25] Leung G, Lim W, Ho L-M, Lam T-H, Ghani A, Donnelly C (2006). Seroprevalence of IgG antibodies to SARS-coronavirus in asymptomatic or subclinical population groups. Epidemiol Infect.

[26] Gierer S, Hofmann-Winkler H, Albuali WH, Bertram S, Al-Rubaish AM, Yousef AA (2013). Lack of MERS coronavirus neutralizing antibodies in humans, eastern province, Saudi Arabia. Emerg Infect Dis.

[27] Müller MA, Meyer B, Corman VM, Al-Masri M, Turkestani A, Ritz D (2015). Presence of Middle East respiratory syndrome coronavirus antibodies in Saudi Arabia: a nationwide, cross-sectional, serological study. Lancet Infect Dis.

[28] Sood N, Simon P, Ebner P, Eichner D, Reynolds J, Bendavid E (2020). Seroprevalence of SARS-CoV-2–Specific Antibodies Among Adults in Los Angeles County, California, on April 10-11, 2020. Jama..

[29] Uyoga S, Adetifa IM, Karanja HK, Nyagwange J, Tuju J, Wanjiku P (2021). Seroprevalence of anti–SARS-CoV-2 IgG antibodies in Kenyan blood donors. Science..

[30] Huff HV, Singh A (2020). Asymptomatic transmission during the coronavirus disease 2019 pandemic and implications for public health strategies. Clin Infect Dis.

[31] Al-Tawfiq JA, Gautret P (2019). Asymptomatic Middle East respiratory syndrome coronavirus (MERS-CoV) infection: extent and implications for infection control: a systematic review. Travel Med Infect Dis.

[32] Ali M, Shah STH, Imran M, Khan A (2020). The role of asymptomatic class, quarantine and isolation in the transmission of COVID-19. J Biol Dyn.

[33] Moriyama M, Hugentobler WJ, Iwasaki A (2020). Seasonality of respiratory viral infections. Ann Rev Virol.

[34] Khan S, Nakajima R, Jain A, De Assis RR, Jasinskas A, Obiero JM (2020). Analysis of serologic cross-reactivity between common human coronaviruses and SARS-CoV-2 using coronavirus antigen microarray. BioRxiv..

[35] Edridge AW, Kaczorowska J, Hoste AC, Bakker M, Klein M, Loens K (2020). Seasonal coronavirus protective immunity is short-lasting. Nat Med.

[36] Boivin G, Hardy I, Tellier G, Maziade J (2000). Predicting influenza infections during epidemics with use of a clinical case definition. Clin Infect Dis.

[37] van den Worm SH, Eriksson KK, Zevenhoven JC, Weber F, Züst R, Kuri T (2012). Reverse genetics of SARS-related coronavirus using vaccinia virus-based recombination. PLoS One.

[38] Hasony HJ, Macnaughton MR (1982). Prevalence of human coronavirus antibody in the population of southern Iraq. J Med Virol.

[39] Gorse GJ, Patel GB, Vitale JN, O'Connor TZ (2010). Prevalence of antibodies to four human coronaviruses is lower in nasal secretions than in serum. Clin Vaccine Immunol.

[40] Woo PC, Lau SK, Huang Y, Yuen K-Y (2009). Coronavirus diversity, phylogeny and interspecies jumping. Exp Biol Med.

[41] Mimbe DE, Byarugaba D, Erima B, Mworozi E, Millard M, Tugume T (2018). Viral causes of Influenza Like Illness in Uganda, 2008 to 2017. Online J Public Health Inform.

[42] Cao W-C, Liu W, Zhang P-H, Zhang F, Richardus JH (2007). Disappearance of antibodies to SARS-associated coronavirus after recovery. N Engl J Med.

[43] Greenberg SB (2016). Update on human rhinovirus and coronavirus infections. Seminars in respiratory and critical care medicine.

[44] Shao X, Guo X, Esper F, Weibel C, Kahn JS (2007). Seroepidemiology of group I human coronaviruses in children. J Clin Virol.

[45] Gaunt ER, Hardie A, Claas EC, Simmonds P, Templeton KE (2010). Epidemiology and clinical presentations of the four human coronaviruses 229E, HKU1, NL63, and OC43 detected over 3 years using a novel multiplex real-time PCR method. J Clin Microbiol.

[46] Principi N, Bosis S, Esposito S (2010). Effects of coronavirus infections in children. Emerg Infect Dis.

[47] Hendley JO, Fishburne HB, Gwaltney JM (1972). Coronavirus infections in working adults: eight-year study with 229 E and OC 43. Am Rev Respir Dis.

[48] Hisali E, Birungi P, Buyinza F (2011). Adaptation to climate change in Uganda: evidence from micro level data. Glob Environ Chang.

[49] Smuts H (2008). Human coronavirus NL63 infections in infants hospitalised with acute respiratory tract infections in South Africa. Influenza Other Respir Viruses.

[50] Owusu M, Annan A, Corman VM, Larbi R, Anti P, Drexler JF, Agbenyega O, Adu-Sarkodie Y, Drosten C (2014). Human coronaviruses associated with upper respiratory tract infections in three rural areas of Ghana. PLoS One.

[51] Sipulwa LA, Ongus JR, Coldren RL, Bulimo WD (2016). Molecular characterization of human coronaviruses and their circulation dynamics in Kenya, 2009–2012. Virol J.

[52] Lu R, Yu X, Wang W, Duan X, Zhang L, Zhou W (2012). Characterization of human coronavirus etiology in Chinese adults with acute upper respiratory tract infection by real-time RT-PCR assays. PLoS One.

[53] Byarugaba DK, Ducatez MF, Erima B, Mworozi EA, Millard M, Kibuuka H (2011). Molecular epidemiology of influenza A/H3N2 viruses circulating in Uganda. PLoS One.

[54] Audi A, AlIbrahim M, Kaddoura M, Hijazi G, Yassine HM, Zaraket H (2020). Seasonality of respiratory viral infections: will COVID-19 follow suit?. Front Public Health.

[55] Tso FY, Lidenge SJ, Pena PB, Clegg AA, Ngowi JR, Mwaiselage J (2021). High prevalence of pre-existing serological cross-reactivity against severe acute respiratory syndrome coronavirus-2 (SARS-CoV-2) in sub-Saharan Africa. Int J Infect Dis.

